# Inclusion of Sunflower Oil in the Bovine Diet Improves Milk Nutritional Profile

**DOI:** 10.3390/nu11020481

**Published:** 2019-02-25

**Authors:** Márcia S. V. Salles, Léa F. D’Abreu, Luiz Carlos R. Júnior, Marcelo C. César, Judite G. L. Guimarães, Julio G. Segura, Cintia Rodrigues, Marcus A. Zanetti, Karina Pfrimer, Arlindo Saran Netto

**Affiliations:** 1 Animal Science Institute (IZ), Ribeirão Preto, SP CEP: 14030-670, Brazil; lcromajr@gmail.com; 2 Faculty of Animal Science and Food Engineering (FZEA), University of São Paulo, Pirassununga, SP CEP: 13635-900, Brazil; lea.fdabreu@gmail.com (L.F.D.); mccesar@usp.br (M.C.C.); julagui@usp.br (J.G.L.G.); guerrasegura@hotmail.com (J.G.S.); cii-rodrigues@hotmail.com (C.R.); mzanetti@usp.br (M.A.Z.); saranetto@usp.br (A.S.N.); 3 Faculty of Medicine of Ribeirão Preto, University of São Paulo, Ribeirão Preto, SP CEP: 14049-900, Brazil; kpfrimer@fmrp.usp.br

**Keywords:** antioxidants, dairy cattle, nutrition, oxidative stability, sensory analysis

## Abstract

Milk and its derivatives are important foods that contribute to daily nutrient requirements and improve consumers’ health. This study evaluated the effects of supplementing the diet of lactating dairy cows with sunflower oil (SFO), selenium, and vitamin E on the milk’s fatty acid profile and fat oxidative stability as well as the acceptability of the milk by consumers. For this purpose, 32 Jersey dairy cows were allocated to four treatment groups for 60 days, as follows: C (control diet); A (3.5 mg/kg DM (dry matter) organic selenium + 2000 IU vitamin E/cow per day); O (4% SFO DM); OA (equal doses of A and O treatments). The inclusion of SFO decreased the contents of 10:0, 10:1, 11:0, 12:0, 12:1, 14:0, and 9c-14:1 fatty acids as well as odd- and branched-chain fatty acids (13:0, iso 13:0, anteiso 13:0, 15:0, iso 15:0, and 17:0). There was also a tendency for 8:0 and 16:0 fatty acid concentrations to decrease when SFO was included in the cows´ diet. SFO decreased the concentration of 10:0 to 15:0 fatty acids in milk. The sum of the conjugated linoleic acids (CLAs), conjugated alpha-linolenic acid intermediates (CLnAs; 18:3 ω6 + 18:3 ω3), and 22:0 fatty acids in milk tended to increase, and there were significant increases in 18:0 and 9c11t-18:2 with SFO. In terms of the effects of SFO on the health-related lipid indices, the atherogenicity index tended to decrease and h/H tended to increase. When cows were supplemented with antioxidants, the concentration of 20:2 fatty acids decreased, the 6 + 7 + 8 + 9t-18:1, 16t-18:1, 20:0, 22:2, and 24:0 fatty acid concentrations increased, and there was a trend for the 22:1 ω9 fatty acid concentration to increase with antioxidants plus oil. There was a tendency for ω6 fatty acids and ω6/ω3 to increase with milk treated with antioxidants plus oil. The oxidative stability of milk was not influenced by the presence of SFO or antioxidants in the diet of dairy cows. Consumers desired the color and mouthfeel of the milk that was treated with SFO. Cows fed with 4% sunflower oil produced milk with an improved fatty acid profile for human nutrition, containing a higher CLA content and an improved ratio of hypocholesterolemic and hypercholesterolemic fatty acids, without increasing the milk’s susceptibility to oxidation. The milk was also rated as being more acceptable by consumers.

## 1. Introduction

The high nutritional value of milk has been recognized since the dawn of civilization, and milk is widely considered to be an essential food that is important for mammals’ proper growth and development and aids in the prevention of chronic diseases such as osteoporosis [1], hypertension [2,3]), and cancer [4,5]. However, due to their high content of saturated fatty acids (SFA) [6], dairy products are often labeled as villains in the human diet. However, there are already papers in the literature that suggest that milk and its derivatives are important components of a healthy dietary pattern and that the health of consumers is guaranteed by regular consumption of these foods [7].

It has been demonstrated that the inclusion of unsaturated vegetable oils in the diet of lactating dairy cows can reduce the proportion of SFA in milk fat substantially, while the contents of unsaturated fatty acids (UFA) and conjugated linoleic acid (CLA) increase [8,9].

The term CLA describes a collection of geometrical and positional isomers of linoleic acid with conjugated double bonds [10] that are considered important promoters of human health due to their potential anticarcinogenic, antiatherogenic, and antidiabetic effects [11]. Moreover, CLA is seen as a potent stimulator of the immune response and are also capable of reducing the accumulation of body fat [12].

Notwithstanding all the possible benefits for human health, increasing UFA concentration in milk is a major disadvantage for the dairy industry, since it predisposes milk fat to oxidation [13], which lead to the development of an off-flavor and off-odor developments [14], causing a loss in the organoleptic and nutritional quality of dairy products and reducing their acceptability by consumers.

To minimize the occurrence of lipid oxidation in milk, dietary inclusion of antioxidants seems to be very efficient [15]. Supplementation with Se increases the blood level of glutathione peroxidase in dairy cows [16], an enzyme that catalyzes the reduction of hydrogen peroxide, inhibiting lipid peroxidation [17]. Vitamin E, in turn, scavenges free radicals, which keeps the lipid hydroperoxide level low in cells and tissues [18].

Based on the aspects mentioned above, we hypothesized that dietary supplementation of lactating dairy cows with sunflower oil (SFO), selenium, and vitamin E would increase the level of CLA in their milk in response to the high concentration of linoleic acid in the diet and the protection offered by the antioxidants against fat oxidation. The present trial was designed to evaluate the effects of the inclusion of sunflower oil, Se, and vitamin E in the diet of dairy cows on the milk fatty acid (FA) profile, the fat oxidative stability, and the sensory characteristics, and consumers’ acceptability of the milk.

## 2. Materials and Methods

This project was approved by the Ethics Committee on Animal Experimentation of the Biological Institute under the protocol number CETEA-IB 113/11.

### 2.1. Study Design

The experiment was carried out for 60 days at the Animal Science Institute (IZ), Ribeirão Preto City, Sao Paulo State, Brazil (21° 10’41.08 "S and 47° 50’45.43" W), with the first fourteen days being an adaptation period. The maximum and minimum temperature averages recorded in the period were 31.6 °C and 18.0 °C, respectively (mean rainfall: 3.50 mm).

The experimental design was a complete randomized block design, with cows distributed at the beginning (up to 100 days), medium (100–180 days), and end (>180 days) of milking. The experiment was conducted with 32 Jersey dairy cows, with a mean live weight of 365.50 ± 42.05 kg and average initial milk yield of 11.02 ± 2.17 kg/day, randomly allocated to individual stalls of 5.2 × 2.6 m (6.76 m^2^ of shade) in the following treatment groups: (1) control concentrate (C); (2) antioxidants: concentrate with supplementation of 3.5 mg kg^−1^ of organic Se in dry matter (DM), and 2000 IU vitamin E (A); (3) SFO: concentrate with the addition of 4% kg^−1^ of sunflower oil in DM (O); and (4) antioxidants + sunflower oil: concentrate with supplementation of 3.5 mg kg^−1^ of organic selenium plus 2000 IU vitamin E and 4% kg^−1^ of sunflower oil in DM (AO).

Cows were fed a total mixed ration (TMR) containing 50% roughage (42% corn silage and 8% coast-cross hay) and 50% concentrate based on dry matter. Diets were formulated to reach the nutritional requirements recommended by the National Research Council [19] (Jersey in lactation, body weight: 400 kg, milk production: 15 kg/day, dry matter intake: 14.19 kg/day or 3.55% of body weight). The source of selenium used was organic selenium (Alkosel, Lallemand®, Montréal, Canada), and the source of vitamin E used was alpha tocopherol acetate; both were mixed with the concentrate.

The supplementation of selenium and vitamin E above the requirements of cows, but below toxic levels, was done with the aim of biofortifying milk for human consumption. The ingredients and chemical compositions of the diets are given in Table 1.

The average dry matter intakes during the experiment were 10.53, 9.53, 10.40, and 9.74 kg/day for C, A, O, and OA treatments, respectively.

### 2.2. Measurements and Sample Collection

Throughout the experimental phase, water and food were provided ad libitum. Control of food intake was performed daily. Cows were milked twice daily (8:00 h and 16:00 h) and milk production (kg/day) was measured during the entire experimental period. Milk samples (morning and afternoon) were taken using an automatic collector once a month from each cow to determine the FA profile and during the last seven days to test the milk’s oxidative stability and to conduct the sensory evaluation.

### 2.3. Feed Assay

Daily samples of the feed and orts were collected and pooled over 2-week periods; they were then mixed and sub-sampled for subsequent chemical analysis. The samples were dried at 55 °C in a forced-air oven for 72 h and grounded with a 1 mm screen mill (FZ102, Shanghai Hong Ji instrument Co., Ltd., Shanghai, China). DM (AOAC, 950.15), ash (AOAC, 942.05), ether extract (EE; AOAC, 920.39), crude protein (CP; AOAC, 984.13), acid detergent fiber (ADF), and lignin (AOAC, 973.18) were analyzed in the feed offered and in samples of orts according to the methods described by the Official Methods of Analysis of AOAC International [20]. The neutral detergent fiber (NDF) content was determined according to the methodology using α-amylase and without the addition of sodium sulphite [21] using the Ankon® system (ANKOM Technology, Macedon NY, USA).

### 2.4. Milk Fatty Acid Assay

To determine the milk FA profiles, milk samples from each cow were initially centrifuged (17,800 × g, 20 min, 8 °C) to separate the fat. From the supernatant cream, 400 mg was transferred to a glass test tube, and fat extraction was conducted [22]. Subsequently, methylation was performed using a methanolic sodium methoxide solution [23]. The separation of fatty acids was achieved by gas chromatography (Trace Model 2000, Thermo Finnigan®, Waltham, MA USA) using a SP-2560 fused silica capillary column (100 × 0.25 × 0.2 mm) and a flame-ionization detector. Hydrogen was used as a carrier gas (flow rate: 1 mL/min). Injector and detector temperatures were set at 250 °C and 300 °C, respectively. The sample injection rate (split mode) was 25:1. Air flow was adjusted to 460 mL/min, and nitrogen flow (auxiliary gas) was 30 mL/min. The initial temperature was 70 °C, increasing to 175 °C after the first injection, and this was then maintained for 27 minutes. Finally, the temperature was raised to 215 °C (4 °C/min) and maintained for 21 minutes. Certified standard butter fat (CRM-164, Commission of the European Communities, Community Bureau of Reference, Brussels, Belgium) was used to determine the recovery of FA and to calculate correction factors. Concentrations of fatty acids are expressed as g/100 g of the total fatty acid concentration.

The nutritional quality of the lipid fraction was assessed using the atherogenicity index:

AI = (C12:0 + (4 × C14:0) + C16:0)/(ΣMUFAs) + Σω6 + Σω3)

and thrombogenicity index [24]:

TI = (C14:0 + C16:0 + C18:0)/[(ΣMUFAs × 0.5) + (0.5 × Σω6) + (3 × Σω3) + (Σω3/Σω6)]

and the ratio of hypocholesterolemic to hypercholesterolemic fatty acids [25]:

h/H = [(C18:1 cis-9 + C18:2 ω6 + C18:3 ω3 + C20:5 ω3 + C22:6 ω3)/(C14:0 + 16:0)]

### 2.5. Determination of Milk Oxidative Stability 

The peroxide index (PI) and thiobarbituric acid reactive substances (TBARS) analysis were performed to determine the formation of primary and secondary products, respectively, during milk fat oxidation. For PI determination, milk fat was extracted by centrifugation [26]. PI measurement was based on the oxidation of ferrous ions to ferric ions, a reaction that is catalyzed by peroxides [27]. The entire procedure was carried out in a dark room and was repeated on the same samples 24 and 96 hours after the first analysis.

TBARS analysis was conducted according to the method based on the reaction between thiobarbituric acid and the products of hydroperoxide decomposition, mainly malondialdehyde, forming a red color complex [28]. The test was repeated on the same samples 24 and 96 hours after the first analysis.

### 2.6. Sensory Evaluation

The research project that this study originated from was submitted and approved (Opinion number 212.367) by the Research Ethics Committee of the School of Animal Science and Food Engineering (FZEA), University of São Paulo (USP). Milk acceptability was determined by assessing the level of acceptance of color, odor, flavor, and mouthfeel using a nine-point hedonic scale (where 1 = really disliked, 5 = not liked nor disliked, 9 = really liked) [29]. Untrained assessors (*n* = 60) were recruited from students (18 to 25 years) and staff at the FZEA to perform both tests. As milk consumption is prevalent at all ages in Brazil, the sole requirement for participation was to be a milk consumer. Prior to sensory evaluation, the milk was pasteurized (72 °C for 30 min) and standardized to 3% fat, but it was not homogenized. Milk was filled into 1 L sterile plastic containers that were hermetically sealed and identified in accordance with the dietary treatment. Milk samples of approximately 50 mL were served at 7 °C after manual mixing. Samples were coded with a 3 digit number and were randomly presented to assessors. Consumer studies usually involve a minimum of 100 participants [30]. Taking this number into account, the low number of assessors that performed the sensory test (*n* = 60) may be regarded as a limitation of our study.

### 2.7. Statistical Analysis

All statistical analyses were performed using SAS (Cary, Carolina do Norte, EUA) [31], version 9.1. Data for milk fatty acid profiles and milk fat oxidative stability were analyzed in a completely randomized design in a 2 × 2 factorial using PROC MIXED, SAS (Cary, NC, USA). Milk fat oxidative stability was analyzed with a repeated measures analysis. Oil, antioxidants, and the interaction between oil and antioxidants were considered fixed effects, and the cow was designated as a random effect. 

The results of sensory analysis were processed by analysis of variance (ANOVA), and Tukey’s test was carried out to compare the mean values of the acceptability test for the different milk produced. Additionally, Dunnett’s test was included to analyze the differences between the control test and other treatment groups. Significance was declared at *p* < 0.05, and a tendency towards significance was declared at *p* ≤ 0.10. 

## 3. Results 

### 3.1. Milk Fatty Acid Composition

The effects of diets on the milk FA profile are shown in Table 2; Table 3. Sunflower oil supplementation decreased (*p* < 0.05) the contents of 10:0, 10:1, 11:0, 12:0, 12:1, 14:0, and 9c-14:1 fatty acids as well as odd- and branched-chain fatty acids (OBCFAs = 13:0, iso 13:0, anteiso 13:0, 15:0, iso 15:0, and 17:0). There was also a tendency for the concentration of 8:0 and 16:0 fatty acids to decrease following oil supplementation in the cows´ diet. Although oil supplementation decreased the concentration of some short and medium chain fatty acids (SMCFAs), it did not change the total amounts of saturated (*p* = 0.184), unsaturated (*p* = 0.173), monounsaturated (*p* = 0.182), or polyunsaturated fatty acids (*p* = 0.109) in milk (Table 3). 

The concentrations of the fatty acids 6 + 7 + 8 + 9t-18:1 (*p* = 0.031), 10 + 11 + 12t-18:1 (*p* < 0.0001), 15c-18:1 (*p* = 0.023), and 16t-18:1 (*p* < 0.0001) in milk were increased following sunflower oil supplementation in the cows’ diet.

The concentration of 22:0 fatty acids (0.011 and 0.007 for with and without oil, *p* = 0.065) tended to increase with sunflower oil supplementation, and the concentrations of stearic (18:0, *p* = 0.009) and rumenic acid (9c, 11t-18:2, *p* < 0.0001) also increased when cows were supplemented with oil.

The sum of CLA and CLnA tended to increase in milk (*p* = 0.093, Table 3) following oil supplementation in the cows’ diet.

Following oil supplementation, the health-related lipid indices tended to include a decreased AI (*p* = 0.066) and an increased h/H (*p* = 0.084).

Dietary antioxidant supplementation did not induce many changes in milk fatty acids, and when it did, it was in the long chain fatty acid concentration, namely, a decrease in 20:2 fatty acids (0.0004 and 0.0011 for with and without antioxidant, *p* = 0.020).

**Figure 1:**
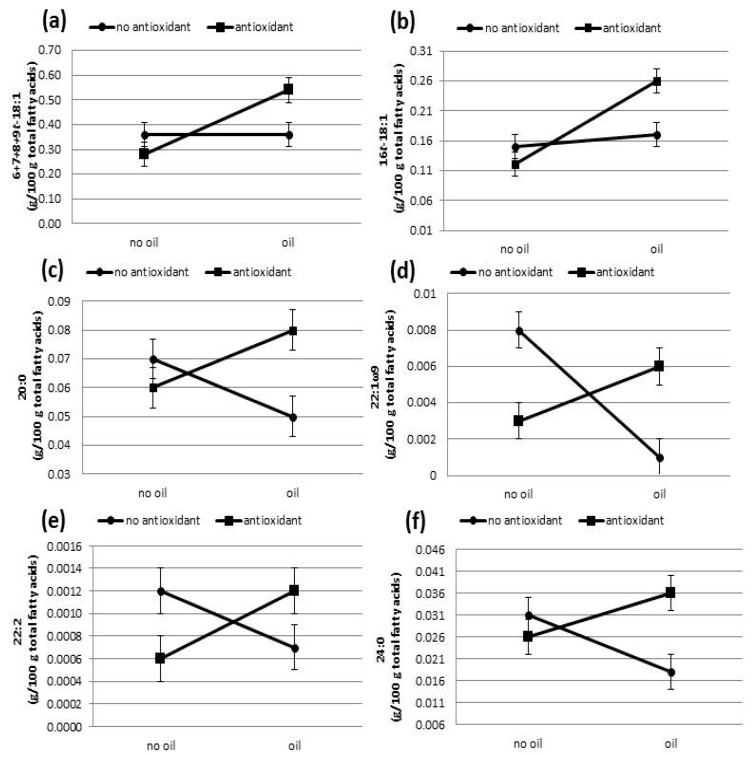
Results for the effect of the interaction between oil and antioxidant supplementation on the milk fatty acid profile for cows fed sunflower oil, vitamin E, and selenium: (**a**), *p* = 0.032, SEM = 0.20; (**b**) *p* = 0.003, SEM = 0.003; (**c**) *p* = 0.004, SEM = 0.004; (**d**) *p* = 0.076, SEM = 0.001; (**e**) *p* = 0.036, SEM = 0.012; (**f**) *p* = 0.026, SEM = 0.004.

There was an effect of the interaction between oil and oxidant supplementation for 6 + 7 + 8 + 9t-18:1 (*p* = 0.032), 16t-18:1 (*p* = 0.003), 20:0 (*p* = 0.004), 22:2 (*p* = 0.036), and 24:0 (*p* = 0.026) fatty acids in milk and a trend for 22:1ω9 (*p* = 0.076, Figure 1). All of these fatty acids had increased concentrations in milk when the cows’ diet was supplemented with antioxidants along with oil.

**Figure 2:**
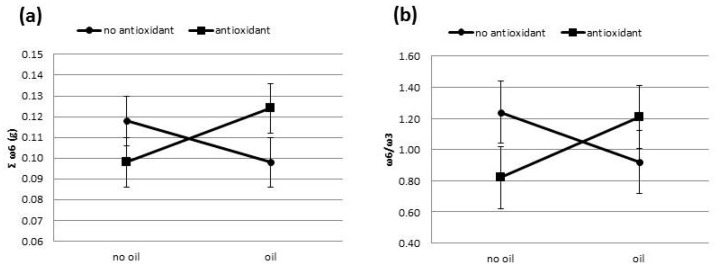
Results for the effect of the interaction between oil and antioxidant supplementation on milk Σ omega-6 fatty acids and Σ omega-6/Σ omega-3 for cows fed sunflower oil, vitamin E, and selenium: (**a**), *p* = 0.081, SEM = 0.012; (**b**), *p* = 0.090, SEM = 0.20.

A similar effect was also found for the sum of ω6 fatty acids (*p* = 0.081) and the relationship between ω6 and ω3 fatty acids (*p* = 0.090), where there was a tendency for the values of these variables to increase following antioxidant supplementation in the presence of the oil (Figure 2).

### 3.2. Oxidative Stability of Milk Fat and Sensory Evaluation of Milk

The mean TBARS value (absorbance at 532 nm) for all dietary treatments was 0.11, and the SEM was 0.01. A time effect was observed for TBARS, which was higher (*p* < 0.0001) at 96 hours (TBARS = 0.09, 0.09, and 0.15, SEM = 0.01 for 0, 24, and 96 days of the experiment, respectively).

The mean peroxide index values (mEq O_2_/kg fat) for treatments with sunflower oil, without sunflower oil, with antioxidants, and without antioxidants were 0.35, 0.38, 0.30, and 0.42 (SEM = 0.06), respectively. There were no effects of time and no interaction between oil and antioxidants (*p* > 0.05).

The consumer panel scores for milk showed than following oil supplementation in the cows’ diet, the perception of color changed (*p* = 0.012), and there was a tendency for the mouthfeel to change (*p* = 0.100, Table 4). The addition of antioxidants in the diets of cows tended to alter the perception of odor in the milk (*p* = 0.086), and there was a significant interaction between oil and antioxidant supplementation for the perception of milk color (6.56, 5.73, 6.51 and 7.03, SEM = 0.25, for the C, A, O and OA treatments, respectively, *p* = 0.006).

## 4. Discussion

This project was carried out concomitantly with a nutrition and human health trial, in which milk from this experiment was pasteurized and offered to elderly people aged over 70 years. Selenium and vitamin E doses were stipulated in the animal feed with the aim of increasing their concentrations in milk to reach the requirements of these nutrients for the elderly. The doses of these antioxidants in animal feed were above the required level, but below toxic doses. We did not intend to change the other parameters of milk composition (protein, lactose, and fat), but only aimed to increase the levels of selenium and vitamin E and to modify the profile of fatty acids in milk. The results of milk performance and composition will be published in another scientific article, but in summary, milk production was not affected by the various treatments, and only supplementation with sunflower oil tended to decrease the protein content in milk (*p* = 0.099, 32.10 and 32.87 g/kg with oil and without oil respectively).

### 4.1. Milk Fatty Acid Composition

The relationship between smaller proportions of 10:0, 12:0, and 14:0 fatty acids and dietary supplementation with unsaturated oils has been reported by other authors [32]. The inclusion of lipid sources rich in long chain UFA in the diet of dairy cows is often characterized by inhibition of the de novo synthesis of short and medium chain fatty acids (SMCFAs) in the mammary glands [6].

The synthesis of OBCFAs in the mammary glands might also be affected by high levels of UFA in the diet of dairy cows, which could explain the lower proportion of these fatty acids in the milk of cows fed sunflower oil [33]. In a previous work, researchers supplemented grazing dairy cows with canola oil, sunflower oil, and linseed oil (0.5 kg/day) and also observed a decrease in OBCFAs in the milk [34].

Since SMCFAs (C12:0–C16:0) are considered hypercholesterolemic [35], and medium chain fatty acids (C6 to C12) from milk induce angiopoietin-like 4 (ANGPTL4) gene expression, which is responsible for the activation in the human colon cancer cell line [36], producing milk with a reduced content of these fatty acids might be interesting for the dairy industry. On the other hand, the medium chain fatty acids (C8 to C12) in milk are associated with beneficial effects that are linked to metabolic activities, such as risk reduction in the development of characteristics of the metabolic syndrome (dyslipidemia, hypertension, obesity, and glucose intolerance, where insulin resistance is the central phenomenon and its co-occurrence is associated with a higher risk of cardiovascular disease) [37].

Previous studies have demonstrated that supplementation of dairy cows with unsaturated oils increases the content of trans-18:1 fatty acids in milk fat [38]. The results of the present study are consistent with this pattern. Milk concentrations of the fatty acids 6 + 7 + 8 + 9t-18:1, 10 + 11 + 12t-18:1, 15c-18:1, and 16t-18:1 increased following supplementation with sunflower oil. Jenkins and Havartine observed that the high dietary supply of UFA exceeds ruminal capacity to biohydrogenate trans-18:1 fatty acids to stearic acid, promoting the accumulation of specific intermediates in the rumen which are then incorporated into the milk [39].

The concentration of 22:0 fatty acids tended to increase following supplementation with sunflower oil, and concentrations of stearic and rumenic acids (9c, 11t-18:2) also increased when cows were supplemented with oil. These fatty acids (stearic and rumenic) are important products of, respectively, the complete and incomplete biohydrogenation of 18C polyunsaturated fatty acids (PUFAs); therefore, these results agree with what was expected for cows supplemented with sunflower oil (rich in ω6 PUFAs).

The sum of CLA and CLnA tended to increase in milk following oil supplementation in the cows’ diet. Potential benefits to human health have attracted researchers’ attention towards the development of effective nutritional strategies to increase the CLA content in the fat of ruminant-derived foods [12]. CLA isomers are formed during ruminal biohydrogenation of UFA or via the action of the ∆9-desaturase enzyme, which is present in the mammary glands, on 11t-18:1, another biohydrogenation intermediate [10]. Oils rich in linoleic acid are considered excellent CLA sources. In this trial, we observed an increase of 0.24 g/100 g of 9c, 11t-18:2, the main CLA isomer, and 0.33 g/100 g of ∑CLnA, CLA for cows fed sunflower oil. A review on the components of milk in human health reported that CLA has demonstrated the ability to prevent different types of cancer, hypertension, atherosclerosis, and diabetes as well as the ability to improve immune function and body composition in in vitro and in vivo studies [37]. 

Although there are 28 different CLA isomers, the health benefits are attributed mainly to two of these: cis-9, trans-11 CLA and trans-10, cis-12 CLA. In addition, the authors reported that there are small amounts of conjugated alpha-linolenic acid intermediates (CLnA) in milk fat that also have anticancer properties [37].

The bioaccessibility of cis-9, trans-11 18:2 was 87.0 ± 3.4% in a dynamic in vitro gastrointestinal model of lactating dairy cows fed safflower oil, and the bioaccessibility was higher for unsaturated FAs than for their saturated counterparts, and the degree of saturated FA absorption decreased with chain length [40]. In another study, the authors carried out an in vitro study of the bioavailability of fatty acids in cow’s milk containing conjugated linoleic acids produced in vivo or added in synthetic form and found that the bioavailability of 9c, 11t-18:2 in milk containing 3.25% fat reached 73%, 89%, 85%, and 71% for the control, and for cows fed 4% sunflower oil in the diet, enriched synthetic free fatty acids, and synthetic triacylglycerol-enriched milk, respectively.^32^ Therefore, 9c, 11t-18:2 was more available in enriched milk by feeding the cow and confirming that CLA is efficiently absorbed in the human small intestine and could therefore confer the alleged health benefits [41].

A review of the aspects of nutritional milk fat globules showed that the polar lipids are anticholesterolemic, reduce the number of aberrant crypt foci and adenocarcinomas, shift the tumor type of malignant tumors to benign, protect the liver from fat and cholesterol induced steatosis, suppress gastrointestinal pathogens, and assist in the maturation of the neonatal intestine and myelination of the developing central nervous system [42]. 

In terms of the health related lipid indices, following oil supplementation, the AI tended to decrease and h/H tended to increase. The lactation stage and genotype affected the fatty acid profile in terms of the milk’s health properties [43]. These authors found higher values of AI in cow’s milk at the later stages of lactation (5.13) and lower values in the middle of lactation (4.08) and emphasized the importance of having low AI in milk for human consumption. The h/H index is the opposite of TI, because the higher the h/H is, the better nutrition is, and the lower the risk of cardiovascular disease is. A high plasma cholesterol level and the occurrence of coronary heart disease are strongly interrelated, and dietary supplementation with polyunsaturated fatty acids (PUFAs) is associated with a reduction in the incidence of occlusive vascular diseases, including atherosclerosis and thrombosis [44].

When antioxidants were supplemented with the oil, there were increased concentrations of 6 + 7 + 8 + 9t-18:1, 16t-18:1, 20:0, 22:2, 24:0, and 22:1ω9 fatty acids in milk. A similar effect was also found for the sum of ω6 fatty acids and the relation between ω6 and ω3, where there was a tendency for the values of these variables to increase following antioxidant supplementation in the presence of the oil. A study about fatty acids and cardiovascular disease showed that there are significant interactions between fatty acids, and the polyunsaturated/saturated ratio must be in the range between 0.5 and 1, and the ω6/ω3 ratio between 2 and 5 [45]. Analyzing this recommendation, the use of milk as food would be exceeding the recommended rate of the correlation between fatty acids due to the presence of a greater amount of saturated fatty acids, but on the other hand, it has an excellent relation between ω6 and ω3 fatty acids. 

A lower ratio of omega-6 to omega-3 fatty acids is desirable for reducing the risk of many chronic diseases that have a high prevalence in western societies as well as in developing countries. For cardiovascular diseases, a ratio of 4:1 was associated with a 70% decrease in total mortality [46]. There was also a lower relation between these omegas that was associated with a reduction in the risk of breast cancer in women [46]. In the present study, the relationship between omega 6 and 3 fatty acids in milk fat was low, almost 1:1, where the best relationship was found following the treatment with antioxidant supplementation without oil (0.098, Figure 2). In this case, the milk produced by the cows in any of the treatments of this experiment could be recommended for consumption without the concern of increasing the proportion between the omegas in the diet.

The major sources of SFA in the diet in most developed countries are milk and dairy products. When oilseeds and plant oils are included in the diet of lactating cows, SFA can be replaced with MUFA and PUFA in milk, offering a mechanism to lower SFA consumption in the human population [47]. In the present experiment, supplementation with sunflower oil in the cows’ diet did not significantly alter SFA/UFA, although the lowest numerical value was for the oil supplementation treatment (2.44 with oil vs. 2.96 without oil), but there were several changes in the amounts of fatty acids within each category of fatty acids, with the decrease of some cholesterolemics and increase of CLA and CLnA.

Understanding the composition of milk fat and how it modifies with different ingredients in the cow diet offers the opportunity to produce milk with a profile of fatty acids that improves the milk nutritional profile and does not cause food restrictions concerning milk and its products. However, commercialization of dedicated supply chains will depend on whether the added costs of production and segregation of modified milk for processing can be recovered at retail [47].

### 4.2. Oxidative Stability of Milk Fat and Sensory Evaluation of Milk

Milk oxidative stability, which was determined by PI and TBARS, was not influenced by the addition of sunflower oil, Se, or vitamin E in the diet of dairy cows. This demonstrates the accumulation of secondary products of lipid oxidation as the storage period of milk is extended. 

Lipid oxidation is a spontaneous and inevitable phenomenon and a major cause of milk deterioration. During this process, double bonds of UFA are attacked by oxidizing compounds, such as free radicals. Although several authors have shown that milk becomes more susceptible to oxidation in response to increased levels of UFA in milk fat [13], according to Rafalowski and collaborators, milk oxidative stability seems to be more strongly related to the antioxidant levels in milk than to the fat composition [48]. 

The lack of significant differences among treatments in this study indicates that all diets provided similar amounts of antioxidants. Being fat-soluble, vitamin E levels are associated with the intake of lipids. In this study, diets with the inclusion of sunflower oil may have provided a sufficient amount of vitamin E to inhibit lipid oxidation in the milk from OA and O treatments, since oil seeds have considerable amounts of vitamin E in their composition. Charmley and collaborators studied the effect of supplemental vitamin E and selenium in the diet on the control of oxidized flavor in milk from Holstein cows and reported that only a proportion of cows (25%) produced milk that was susceptible to oxidation, and there was a trend toward a reduced oxidized flavor when vitamin E was given [49].

Sensory analysis of food products is very important for monitoring food quality and shelf life. Moreover, sensory characteristics play a key role in determining the merchantability of food products.

The hydrolysis of triacylglycerides from milk results in the release of free fatty acids, causing the rancid taste of milk, and the authors compared new and standard methods for the analysis of fatty acids in milk, including the relation with rancid flavor, and found that hexanoic acid (C6) was the one that had a greater correlation with the rancid flavor [50]. In this trial, the C6 concentration in milk fatty acids was not different among treatments.

Consumers desired the color and mouthfeel of the milk originating from the oil treatment, noting that all milks were standardized to have 3% fat. The addition of antioxidants in the diet of cows tended to alter the perception of odor in the milk, whereby the consumers preferred the milk of cows that were exposed to the treatment without antioxidants. 

The color perception of the milk did not change following treatments without antioxidants, but the consumers liked the milk color more when the antioxidants were combined with the oil.

The visual appearance is the feature by which consumers evaluate the quality of the product [51]. Milk color depends on the concentration of natural pigments, such as carotenoids and riboflavin [52], and on the scattering of light by fat globules and casein micelles [53]. 

The type of processing to which milk is subjected can influence its color, particularly its whiteness [54]. Hayes and Kelly observed that homogenized milk becomes whiter [55], which may be related to the increased number of fat globules and a more efficient dispersion of light [56]. Homogenization of milk is a process through which the size of fat globules is reduced, whereas the opposite effect is observed for the surface area of fat globules [57]. In this study, milk was not homogenized before or after heat treatment, explaining the positive perception by consumers regarding the color and mouthfeel of the milk from treatment with oil.

Khanal and collaborators studied the consumer acceptability of conjugated linoleic acid-enriched milk from cows grazing on pasture and suggested that the consumer acceptability attributes of CLA-enriched milk from cows grazing on pasture are similar to those of milk with normal levels of CLA from cows fed a conventional diet [58]. In the present experiment, milk from cows supplemented with sunflower oil was preferred in terms of color and mouthfeel, indicating that it may have good acceptability by consumers in the dairy market.

## 5. Conclusions

Dietary inclusion of Se and vitamin E supplementation had little influence on the fatty acid profile of milk or on lipid peroxidation. Cows fed 4% SFO produced milk with an improved fatty acid profile for human nutrition, which contained a higher CLA content and lower hypercholesterolemic fatty acid content, without increasing the susceptibility of milk to oxidation and improving the acceptability of milk for consumers. 

## Figures and Tables

**Table 1:** Experimental diets and chemical compositions of the experimental diets.

	Treatments ^2^			
Ingredients (%)	C	A	O	OA
Corn Silage	42.00	42.00	42.00	42.00
Coast-Cross Hay	8.00	8.00	8.00	8.00
Corn Meal	20.00	20.00	16.00	16.00
Soybean Meal	18.00	18.00	18.00	18.00
Wheatmeal	4.00	4.00	4.00	4.00
Urea	0.90	0.90	0.90	0.90
Salt	0.50	0.49	0.50	0.49
Mineral Complex ^1^	1.00	1.00	1.00	1.00
Ammonium Sulfate	0.04	0.04	0.04	0.04
Sodium Bicarbonate	0.53	0.53	0.53	0.53
Sunflower Oil ^3^	-	-	4.00	4.00
Selenium (mg/kg)	-	3.50	-	3.50
Vitamin E (UI/day)	-	2000	-	2000
**Nutrients (%MS)**				
Dry Matter (%)	62.39	62.58	62.65	62.69
Crude Protein	20.30	18.71	18.37	20.32
Ether Extract	2.51	2.51	4.27	4.14
Mineral Matter	7.00	8.03	7.94	7.84
Neutral Detergent Fiber	34.87	34.96	34.65	34.74
Acid Detergent Fiber	20.36	20.40	20.79	20.68
N-Neutral Detergent Fiber	9.76	10.25	9.67	9.58
N-Acid Detergent Fiber	7.53	7.81	8.23	7.58
Cellulose	17.05	16.87	17.29	17.19
Lignin	2.85	2.94	2.90	2.92
Energy (cal/g)	4033.38	3997.37	4082.07	4094.70
Hemicellulose	14.50	14.57	13.87	14.07
Calcium (g/kg)	12.33	9.17	9.50	9.24
Phosphorus (g/kg)	4.23	5.70	4.72	5.56
Magnesium (g/kg)	2.58	2.68	2.77	2.56
Sulfur (g/kg)	2.54	3.33	3.00	3.16
Potassium (g/kg)	9.11	9.07	8.85	8.92
Selenium (mg/kg)	0.18	2.98	0.23	3.16
Iron (mg/kg)	680.32	821.13	743.15	813.30
Copper (mg/kg)	36.61	78.86	72.75	89.54
Zinc (mg/kg)	120.66	193.67	176.56	186.61
Manganese (mg/kg)	85.70	112.88	114.59	112.88
α-Tocopherol (mg/kg)	21.22	60.86	26.28	67.52

^1^ Composition per kilogram of the product: S—80 g, Mg—20 g, K—20 g, Mn—1000 mg, Zn—2500 mg, Cu—1500 mg, Co—100 mg, I—80 mg, inorganic Se—20 mg, Ca—180 g, P—90 g, F—max 300 mg. ^2^ Treatments: C—control; A—antioxidants (Se +vitamin E); O—sunflower oil; AO—antioxidants + sunflower oil. ^3^ Sunflower oil fatty acid profile (g/100 g total fatty acids): 6:0, 8:0, and 10:0—not detected; 12:0—up to 0.1; 14:0—up to 0.2; 16:0—5 to 7.6; 16:1—up to 0.3; 17:0—up to 0.2; 17:1—up to 0.1; 18:1—14 to 39.4; 18:2—48.3 to 74; 18:3—up to 0.3; 20:0—0.1 to 0.5; 20:1—up to 0.3; 20:2—not detected; 22:0—0.3 to 1.5; 22:1—up to 0.3; 22:2—up to 0.3; 24:0—up to 0.5; and 24:1—not detected (Codex Alimentarius, 2005).

**Table 2:** Milk fatty acid profiles (g/100 g total fatty acids) for cows fed sunflower oil, vitamin E, and selenium.

Fatty Acids ^1^		Treatment				*p*-Value ^3^		
	Sunflower Oil		Antioxidants					
	With	Without	With	Without	SEM ^2^	O	A	OxA
4:0	2.4285	2.2356	2.2978	2.3563	0.1171	0.2334	0.7278	0.7225
6:0	1.6576	1.6577	1.6790	1.6363	0.1364	0.9999	0.8268	0.6481
8:0	0.7180	0.8504	0.7578	0.8106	0.0498	0.0727	0.4623	0.8241
10:0	1.6891	2.2256	1.8704	2.0444	0.1475	0.0169	0.4142	0.9561
10:1	0.1320	0.1835	0.1526	0.1630	0.0165	0.0374	0.6622	0.2776
11:0	0.0234	0.0343	0.0279	0.0298	0.0034	0.0363	0.7082	0.4225
12:0	2.0386	2.787	2.3090	2.5169	0.1915	0.0108	0.4515	0.9800
12:1	0.0198	0.0313	0.0240	0.0313	0.0038	0.0480	0.5783	0.2086
13:0	0.0654	0.0892	0.0761	0.0785	0.0066	0.0161	0.7995	0.7279
*iso* 13:0	0.0342	0.0424	0.0382	0.0384	0.0024	0.0309	0.9643	0.1628
*anteiso* 13:0	0.0454	0.0717	0.0550	0.0621	0.0091	0.0067	0.4292	0.4260
14:0	7.8408	9.7588	8.3117	9.2880	0.5153	0.0145	0.1934	0.6910
*iso* 14:0	0.1001	0.1095	0.0991	0.1105	0.0012	0.4548	0.3623	0.1158
9*c*-14:1	0.5713	0.7538	0.6378	0.6874	0.0650	0.0420	0.5652	0.1397
15:0	0.9283	1.1315	1.0186	1.0411	0.0464	0.0049	0.7350	0.4644
*iso* 15:0	0.2371	0.3014	0.2695	0.2690	0.0154	0.0069	0.9806	0.9652
*anteiso* 15:0	0.3833	0.4351	0.4088	0.4096	0.0289	0.2184	0.9855	0.1121
16:0	31.1715	36.4695	33.2483	34.3926	1.8373	0.0526	0.6641	0.4625
*iso* 16:0	0.2369	0.2746	0.2472	0.2643	0.0201	0.1987	0.5554	0.1930
9*c*-16:1	1.7331	1.9239	1.7969	1.8601	0.1513	0.3820	0.7708	0.6555
17:0	0.6918	0.8211	0.7749	0.7380	0.0369	0.0085	0.4227	0.9527
*iso* 17:0	0.4675	0.4436	0.4605	0.4506	0.0177	0.3531	0.6967	0.3196
17:1	0.2422	0.2639	0.2503	0.2558	0.0352	0.6684	0.9124	0.9734
18:0	16.3261	12.2372	14.9994	13.5638	1.0331	0.0099	0.3363	0.2094
6+7+8+9*t*-18:1	0.4526	0.3231	0.4114	0.3643	0.0401	0.0316	0.4162	0.0325
10+11+12*t*-18:1	2.2160	1.2102	1.7667	1.6595	0.1547	0.0001	0.6293	0.6053
9*c*-18:1	20.6488	17.5691	19.3948	18.8231	1.7807	0.2338	0.8227	0.8454
11*c*-18:1	1.6563	1.3782	1.5665	1.4680	0.1442	0.1859	0.6340	0.9206
12*c*-18:1	0.8983	0.7623	0.8480	0.8126	0.0756	0.2168	0.7439	0.6975
13*c*-18:1	0.4921	0.4058	0.4662	0.4316	0.0417	0.1573	0.5645	0.7867
15*c*-18:1	0.0516	0.0356	0.0484	0.0388	0.0046	0.0231	0.1577	0.8662
16*t*-18:1	0.2160	0.1370	0.1935	0.1595	0.0127	0.0002	0.0726	0.0039
18:2 ω6	1.5101	1.3900	1.5002	1.4001	0.1110	0.4722	0.5378	0.6989
9*c*, 11*t*-18:2	0.6547	0.4065	0.5381	0.5231	0.0407	0.0002	0.7982	0.7472
18:3ω6	0.0702	0.0626	0.0692	0.0636	0.0070	0.4542	0.5868	0.1229
18:3ω3	0.1060	0.1094	0.1095	0.1059	0.0099	0.8126	0.7991	0.7656
20:0	0.0649	0.0695	0.0732	0.0612	0.0049	0.5121	0.0978	0.0049
20:1	0.0727	0.0751	0.0829	0.0649	0.0079	0.8353	0.1255	0.2813
20:2	0.0008	0.0006	0.0004	0.0011	0.0002	0.4675	0.0205	0.4675
20:3ω6	0.0041	0.0052	0.0041	0.0051	0.0014	0.5885	0.6304	0.2346
20:3ω3	0.0063	0.0058	0.0073	0.0048	0.0011	0.7605	0.1033	0.1620
20:4ω6	0.0368	0.0404	0.0379	0.0393	0.0072	0.7327	0.8903	0.3689
20:5ω3	0.0013	0.0011	0.0008	0.0016	0.0004	0.8343	0.2521	0.5567
21:0	0.0057	0.0056	0.0056	0.0057	0.0008	0.9496	0.9496	0.9496
22:0	0.0140	0.0053	0.0112	0.0081	0.0031	0.0653	0.5123	0.1425
22:1ω9	0.0037	0.0057	0.0047	0.0048	0.0010	0.4667	0.9749	0.0768
22:2	0.0010	0.0009	0.0009	0.0010	0.0001	0.7748	0.7748	0.0360
22:5	0.0205	0.0225	0.0195	0.0235	0.0026	0.6248	0.3284	0.5644
22:6ω3	0.0010	0.0013	0.0012	0.0011	0.0002	0.2976	0.9729	0.9729
23:0	0.0038	0.0043	0.0050	0.0030	0.0008	0.6817	0.1032	0.2487
24:0	0.0272	0.0281	0.0307	0.0246	0.0033	0.8474	0.2153	0.0267
24:1	0.0003	0.0002	0.0004	0.0001	0.0001	0.6506	0.2731	0.9875
Total	99.0340	99.0796	99.0209	99.0927	0.0912	0.7272	0.5838	0.7272

^1^ c = cis; t = trans; ^2^ SEM = standard error of mean; ^3^ O = with sunflower oil vs. without sunflower oil; A = with antioxidants vs. without antioxidants; OxA = interaction between oil and antioxidant supplementation.

**Table 3:** Nutritional quality of the lipid fraction of milk (g/100 g total fatty acids) for cows fed sunflower oil, vitamin E, and selenium.

Fatty Acids	Treatment					*p*-Value ^2^		
	Sunflower Oil		Antioxidants					
	With	Without	With	Without	SEM ^1^	O	A	OxA
∑CLnA, CLA	2.3548	2.0166	2.2424	2.1290	0.1370	0.0932	0.5637	0.5379
ΣSFA	67.2003	71.9411	69.0761	70.0653	2.4490	0.1841	0.7780	0.9559
ΣUFA	31.8327	27.1381	29.9443	29.0265	2.3653	0.1737	0.7865	0.9435
ΣMUFA	29.4058	25.0594	27.6456	26.8197	2.2377	0.1827	0.7967	0.9042
ΣPUFA	2.4268	2.0786	2.2987	2.2068	0.1481	0.1098	0.6654	0.4945
Σω3	0.1146	0.1178	0.1189	0.1135	0.0141	0.8199	0.6976	0.6648
Σω6	0.1112	0.1083	0.1113	0.1082	0.0088	0.8186	0.8095	0.0814
SFA/UFA	2.4365	2.9623	2.5837	2.8150	0.3608	0.3142	0.6553	0.7852
ω6/ω3	1.0686	1.0278	1.0163	1.0801	0.1412	0.8405	0.7530	0.0900
AI	2.4823	3.4360	2.7324	3.1858	0.3504	0.0663	0.3701	0.8428
TI	3.9813	4.6558	4.1268	4.5103	0.6226	0.4518	0.6675	0.6909
h/H	0.5732	0.4067	0.5024	0.4775	0.0653	0.0845	0.7907	0.9733

∑CLnA, CLA = CLnA (18:3 ω6 + 18:3 ω3) + CLA (18:2 ω6 + 9c,11t-18:2); ΣSFA = Σ saturated fatty acids; ΣUFA = Σ unsaturated fatty acids; ΣMUFA = Σ monounsaturated fatty acids; ΣPUFA = Σ polyunsaturated fatty acids; Σω3 = Σ omega-3 fatty acids; Σω6 = Σ omega-6 fatty acids; SFA/UFA = saturated/unsaturated; ω6/ω3 = Σ omega-6/Σ omega-3; AI = atherogenicity index; TI = thrombogenicity index; h/H = ratio of hypocholesterolemic to hypercholesterolemic fatty acids. ^1^ SEM = standard error of mean ^2^ O = with sunflower oil vs. without sunflower oil; A = with antioxidants vs. without antioxidants; OxA = interaction between oil and antioxidant supplementation.

**Table 4:** Open panel scores for the consumer acceptability of milk from cows fed sunflower oil, selenium, and vitamin E.

	Treatment					*p*-Value ^2^		
	Sunflower Oil		Antioxidants					
	With	Without	With	Without	SEM ^1^	O	A	OxA
Color perception	6.77	6.15	6.38	6.54	0.17	0.012	0.522	0.006
Odor	6.46	6.19	6.15	6.50	0.14	0.190	0.086	0.140
Flavor	6.42	6.08	6.07	6.43	0.17	0.169	0.149	0.481
Mouthfeel	6.63	6.25	6.26	6.62	0.16	0.100	0.133	0.352

^1^ SEM = Standard Error of Mean; ^2^ O = with sunflower oil vs. without sunflower oil; A = with antioxidants vs. without antioxidants; OxA = interaction between oil and antioxidant supplementation.
